# Supplementary material for: Patterns of Intron Gain and Loss in Fungi
Source: PLoS Biol. 2004 Nov 30;2(12):e422. doi: 10.1371/journal.pbio.0020422 (PMC532390; doi:10.1371/journal.pbio.0020422)
Supplement: Table S1 — Also available at http://genes.mit.edu/NielsenEtAl/. (4.3 MB ZIP). [file pbio.0020422.st001.zip › NielsenEtAl/html/337.html]

AN4951.1.NCU06284.1.MG05089.1.FG02756.1


```
 CLUSTAL W (1.82) Multiple Sequence Alignments - Introns Inserted


Sequence 1: NCU06284.1	881 aa
Sequence 2: MG05089.1	898 aa
Sequence 3: FG02756.1	902 aa
Sequence 4: AN4951.1	866 aa
Alignment Length: 908 aa
Number Identitical Residues: 525 aa
Alignment Score (without introns) 24230


MG05089.1 	MASVPAPPEDQARLLEDALIAVRQQTQMMRKCLDTPGKLMDALKCC2STLVSELRTSSLG
NCU06284.1	-MSTPAPPEDQARLLEDALIAVRQQSTLMRKCLDTPGKLMDALKCC2STLVSELRTSSLG
FG02756.1 	-MATPAPPEDQARLLEDALVAVRQQTSLMRKCLDTPGKLMDALKCC2STLVSELRTSSLG
AN4951.1  	MATSVAPSEDQSRLLEEALGVVRQQSSMMRKCLETPGKLMDALKCG2STLVSELRTPSLG
          	  :  **.***:****:** .****: :*****:***********  *********.***

MG05089.1 	PKQYYELYMSVFDALRYLSVHLRENHQVNHLADLYELVQYAGNIIPRLYLMITVGTAYMA
NCU06284.1	PKQYYELYMAVFDALRYLSVHLRENHPVNHLADLYELVQYAGNIIPRLYLMITVGTAYMS
FG02756.1 	PKQYYELYMSVFDALRYLSVHLRENHPVNHLADLYELVQYAGNIVPRLYLMITVGTAYMA
AN4951.1  	PKQYYELYMAVFDALRHLSVYLKENHPVNHLADLYELVQYAGNIVPRLYLMITVGTVYMS
          	*********:******:***:*:*** *****************:***********.**:

MG05089.1 	IDAAPVKELMKDMMDMSRGVQHPIRGLFLRYYLSGQARDFLPQGEGDGPEGNLQDSINFV
NCU06284.1	IDGAPVKELMKDMMDMSRGVQHPVRGLFLRYYLSGQARDYLPTGDSEGPEGNLQDSINFI
FG02756.1 	IEDAPVKELMKDMMDMSRGVQHPIRGLFLRYYLSGQARDFLPTTESDGPEGNISDSINFV
AN4951.1  	VEDAPVKEIMKDMMEMSRGVQHPIRGLFLRYYLSGQARDYLPTGTGNGPEGNIQDSINFV
          	:: *****:*****:********:***************:**   .:*****:.*****:

MG05089.1 	LTNFVEMNKLWVRLQHQGHSREREQRTQERKELQLLVGSNLVRLSQLVDLEAYKTAILAP
NCU06284.1	LTNFVEMNKLWVRLQHQGHSRERDQRTQERKELQLLVGNNIVRLSQLVDLPTYKNGILAP
FG02756.1 	LTNFVEMNKLWVRLQHQGHSREREQRIRERKELQLLVGSNIVRLSQLVDLETYKSSILAP
AN4951.1  	LTNFVEMNKLWVRLQHQGPSREREKRMQERRELELLVGSNVVRLSQLVDLDTYKSGILQA
          	****************** ****::* :**:**:****.*:********* :**..** .

MG05089.1 	LLEQVVQCRDVLAQEYLLEV~ITQVFPDEYHLHTLDQFLAAVSRLNPHVNVKAIVIGLMD
NCU06284.1	LLEQVVQCRDVLAQEYLLEV~ITQVFPDEFHLHTLDQFLAAVSRLNPHVNVKGIVIGLMD
FG02756.1 	LLEQVVQCRDVLAQEYLLEV~ITQVFPDEFHLHTLDQFLGAVSRLNPHVNVKAIVIGLMD
AN4951.1  	LLEQVVQCRDVLAQEYLLEV1ITKVFPDEFHLHTLDLLLSAIARLNPHVDLKKIVIGLMD
          	******************** **:*****:****** :*.*::******::* *******

MG05089.1 	RLSDYAERESQNEP-EEDRETMEADAIAELLEKVKIARENAESSGPPPPPPPE----EHK
NCU06284.1	RLSDYAEREAQNEE-GEDRGQLEEEALANLLERVKLVKSAAETEEASAPTP---------
FG02756.1 	RLSEYAERDGQEDK-SEDKAQMEADALAKLLEKVNLLKEIPATPPSDSKPPEEGKSSDSS
AN4951.1  	RLSSYAAREAETSMNAETRKQKEEEAVTKLLENLKVSEASQEK-----------------
          	***.** *:.: . . * :   * :*:::***.::: .    .                 

MG05089.1 	RLEMEDENIARTEADVD---STAETLGQDGQSIADSEATAVNGQDGEGNGEQQEPVKKHR
NCU06284.1	---EAPENGEHAETDVG---STDEPLNAEP-SVAETEATVTNG-------DEVEPAKKHR
FG02756.1 	ESTESPDDTKTPESEDGPTAKTEEPANETAGESSTDDSSTLAESTPSVADTETTAVNGQG
AN4951.1  	-----PKEDATPTQENG----VEQTPTESEEQTKPADEVTANGR-----DEDQKPTS---
          	      .:   .  : .    . :.      .    :  .         . :  ...   

MG05089.1 	GIPENVKLYEIFFGQVKNLVQAQHLPIQDTIALLVSLVNLALNIYPGRLDYVDQVLEYAT
NCU06284.1	GIPENVPLYEIFFGQVKNLVQAQHLPIQDTIALCVSLTTLALNIYPERLDYVDQILDYAH
FG02756.1 	SITEKVQLYEVFFAQVKNLVEAQHLPVPDIIALLVSLCNLALNIYPDRLDYVDQILAYAT
AN4951.1  	--PQDIKLYDIFYEQVVSLIKSRGLPIQDTMALLVSLVNLALNTYPERLEYVDQILQFAT
          	  .:.: **::*: ** .*:::: **: * :** *** .**** ** **:****:* :* 

MG05089.1 	TKVREHANSPDLHSPPAQQSLLALLQAPLKRYVSMFTALALPTYVPLYQAQTYPTRRAVA
NCU06284.1	SKVKEHANSADLHAPPAQQSILALLQAPLKRYVSIFTALALPTYVPLFQSQTYPTRRAVA
FG02756.1 	TKVRENMNNADLHSPPAQQSLLALLQAPLNHYVSTFTALSLPTYVPLFQSQSYPTRRAVA
AN4951.1  	KETAEYTDHADLHAAPTQQNLLHLLIAPLRSYVSVFTALALPHYLPLLSSQSYPTRRSVA
          	.:. *  : .***:.*:**.:* ** ***. *** ****:** *:** .:*:*****:**

MG05089.1 	GEVARTLLRDRIQICTVEQLENVLEILKVLIKEGSHPPQGYPGVAPARQRVMETDETLEE
NCU06284.1	GEVARHLIKNQTHITTTANLENVLEVLKVLIKEGSQAPSGYPGVVQQRGRALETDETLEE
FG02756.1 	GDVVRTLLKNQTKISTTEQLENVLEVLKVLIKEGSQAPQGYPGVSQRR--PVETDETMEE
AN4951.1  	GEIARTLLKDRTLITTTENLDRVLQALRVLIKEGVQQ-GGYPGSQRRG----ESDETIEE
          	*::.* *::::  * *. :*:.**: *:****** :   ****         *:***:**

MG05089.1 	QGWLARIVHLLDSENNDTQFK~LLQMTRKAYGDGNDRIRTTTPPLITAGMKLARRFKARE
NCU06284.1	QGWLARLVHLLQAEDNDTQFR~LLQMTRKAYAEGNERIRTTTPPLITAGLKLARRYKARE
FG02756.1 	QGWLARIVHLLQAEDNDTQFK~LLQMTRKAYSEGNERIRTTTPPLLTACMKLARKFKQRE
AN4951.1  	QGWLARLVHLLQAPENDTQLK0LLQATRKAYLDGNERIRYTFPAIVSSSIRLARKLKSRE
          	******:****:: :****:: *** ***** :**:*** * *.:::: ::***: * **

MG05089.1 	HYDDNWSSQSSALFKFLHSAISTLYARVNGAGAAELSLRLFCACGQTADAAGFEEVAYEF
NCU06284.1	HYDDNWQSQCSALFKFLHSAISTLYTRVNGAGAAELSLRLFCSCGQMADKTEFEEVAYEF
FG02756.1 	HFDDNWETQSNALFKFMHSALSTLYTRVNSSGAAEMALRLFASAGQTADLVGFEEVAYEF
AN4951.1  	HYDDNWQSQSSALYRFMHQCVNNLYQRVN-PGCADLALRLFVMCGEVADQTGFEEFSYEF
          	*:****.:*..**::*:*..:..** *** .*.*:::****  .*: ** . ***.:***

MG05089.1 	FAQAFTVYEEAVSDSKAQFQAVCVVASALHQTRNFGKENYDTLITKCAQHSSKLLRKPDQ
NCU06284.1	FAQAFTVYEEAISDSKAQFQAVCAIASALHRTRNFGKENYDTLITKCAQHASKLLRKPDQ
FG02756.1 	FAQAFTVYEEAVTDSKAQFQAVCVIATALHQTRNFGKENYDTLITKCAQHGSKLLRKPDQ
AN4951.1  	FAQAFTIYEDSISDSRAQFQAVCIIAGALHGTRGFSKENYDTLITKAALHGSKLLKKPDQ
          	******:**::::**:******* :* *** **.*.**********.* *.****:****

MG05089.1 	CRAVYLASHLWWATPIAANGEDENTE0LYRDGKRVLECLQRALRVADSCMETATSIELFV
NCU06284.1	CRAVYLASHLWWATPIASNGETEETE0LYRDGKRVLECLQRALRVADSCMETATSIELFV
FG02756.1 	CRAVYLASHLWWATPIVSNGESEETE0LYRDGKRVLECLQRALRVADSCMETATSIELFV
AN4951.1  	CRAVYLASHLWWVIENPHRGEEDPKN0LYRDGKRVLECLQRALRVADACMDTAVSVELFV
          	************.     .** : .: ********************:**:**.*:****

MG05089.1 	EILDRYVYYFDQRNES0VTTKYLNGLIELIHSNFAGNQQD-SASVEACRKHFQHTLDLIR
NCU06284.1	EILDRYVYYFDQKNES0VTTKYLNGLIELIHSNLAGNQQD-SASVEASRKHFMQTLEIIR
FG02756.1 	EILDRYVYYFDQQNES0VTTKYLNGLIELIHSNLAGNQQD-SASVENSRRHFNQTLENIR
AN4951.1  	EILNRYVYYFDQQNET~VTTKYLNGLIELIHSNLQTDQDEPNPALENPKRHFYRTLEYIR
          	***:********:**: ****************:  :*::...::*  ::** :**: **

MG05089.1 	SKEYEGIVLDPK-
NCU06284.1	SKEYEGIVLTPK-
FG02756.1 	SRQYEGIVLTPN-
AN4951.1  	AREFEGVVTDPRS
          	::::**:*  *.:
```
